# Recommendation for management of patients with their first episode of primary spontaneous pneumothorax, using video-assisted thoracoscopic surgery or conservative treatment

**DOI:** 10.1038/s41598-021-90113-w

**Published:** 2021-05-25

**Authors:** Hsin-Yi Chiu, Yi-Chia Ho, Pei-Chen Yang, Chi-Ming Chiang, Cheng-Chin Chung, Wei-Ciao Wu, Yu-Cih Lin, Chien-Yu Chen, Yu-Chung Wu

**Affiliations:** 1 Center for Evidence-Based Medicine, Department of Medical Education, Taipei Medical University Hospital, Taipei, Taiwan, ROC; 2 School of Medicine, College of Medicine, Taipei Medical University, Taipei, Taiwan, ROC; 3 Department of Orthopaedics Surgery, Cardinal Tien Hospital Ankang Branch, New Taipei City, Taiwan, ROC; 4 Professional Master Program for Artificial Intelligence in Medicine, College of Medicine, Taipei Medical University, Taipei, Taiwan, ROC; 5 Division of Thoracic Surgery, Department of Surgery, Taipei Medical University Hospital, Taipei, Taiwan, ROC; 6 Department of Education and Humanities in Medicine, School of Medicine, College of Medicine, Taipei Medical University, Taipei, Taiwan, ROC; 7 Department of Anesthesiology, School of Medicine, College of Medicine, Taipei Medical University, Taipei, Taiwan, ROC; 8 Department of Anesthesiology, Taipei Medical University Hospital, Taipei, Taiwan, ROC; 9 Graduate Institute of Humanities in Medicine, College of Humanities and Social Sciences, Taipei Medical University, Taipei, Taiwan, ROC; 10 School of Nursing, College of Nursing, Taipei Medical University, Taipei, Taiwan, ROC; 11 Department of Surgery, School of Medicine, College of Medicine, Taipei Medical University, Taipei, Taiwan, ROC; 12 Department of Animal Science and Technology, National Taiwan University, Taipei, Taiwan, ROC; 13 Division of Thoracic Surgery, Department of Surgery, Shuangho Hospital, Taipei, Taiwan, ROC; 14 Department of Medical Education, Chi-Mei Medical Center, Tainan, Taiwan, ROC; 15 Department of Medical Education, National Taiwan University Hospital, Taipei, Taiwan, ROC; 16 Division of Trauma, Emergency Department, Cardinal Tien Hospital Ankang Branch, New Taipei City, Taiwan, ROC

**Keywords:** Respiratory tract diseases, Therapeutics

## Abstract

International guidelines do not recommend surgery for the first episode of primary spontaneous pneumothorax (PSP), except in cases of persistent air leak, hemopneumothorax, bilateral pneumothorax, or occupations at risk. However, these recommendations have been challenged because of a significant reduction in the recurrence rate in emerging studies. We evaluated the rationale of recommendations by systematically reviewing RCTs and observational studies by using the Grading of Recommendations, Assessment, Development, and Evaluations (GRADE) system. We searched articles in PubMed, EMBASE, and Cochrane databases up to August 15, 2020. The primary outcomes were the recurrence rate and complication rate. The secondary outcomes were hospital stay and drainage duration. Nine eligible studies with 1121 patients were retrieved and analyzed. The recurrence rate was lower in the VATS than in conservative treatment with moderate evidence (OR 0.13, 95% CI 0.09 to 0.19, *P* < 0.001, I^2^ = 0%). We did not find significant differences in complication rate (Peto OR 1.17, 95% CI 0.33 to 4.12, *P* = 0.80), hospital stay duration (MD − 0.48 days, 95% CI − 2.84 to 1.87, *P* = 0.69, very low evidence), and in drainage duration (MD − 3.99 days, 95% CI − 9.06 to 1.08, *P* = 0.12, very low evidence) between the two groups. Our results would suggest VATS treatment as a weak recommendation for patients with the first episode of PSP, based on our systematic review of the current evidence by using the GRADE system, indicating that different treatments will be appropriate for different patients and that patients’ values and preferences should be incorporated through shared decision making.

Trial REGISTRY: PROSPERO; No.: CRD42020162267.

## Introduction

Primary spontaneous pneumothorax (PSP) is a significant global health concern that commonly affects young people who do not have clinically apparent lung disease. The annual incidence rates of PSP are 18–28 per 100,000 in men and 1.2–6 per 100,000 in women^1^. PSP typically results from the rupture of subpleural blebs or bullae^2^. According to international guidelines, conservative treatments, such as observation, simple aspiration, and chest tube treatment, are established as the first-line treatments for the first episode of PSP in patients^3,4^. However, the most substantial problem of PSP under conservative management is the variable recurrence rate ranging from 14 to 50% within one to 5 years in patients^4–7^.

With advancements in surgical technology, video-assisted thoracoscopic surgery (VATS) has become the mainstream avenue of thoracic surgery. Compared with traditional open thoracotomy, VATS has the advantages of smaller operative wound size and less hospitalization time. Therefore, studies have investigated the effectiveness and safety of performing VATS for the first episode of PSP^8,9^ despite international guidelines not recommending surgery for initial pneumothorax except in cases of persistent air leak, hemopneumothorax, bilateral pneumothorax, or for people with at-risk occupations^3,4,10^. The optimal management of patients during their first episode of PSP has remained debatable.

Clinical guidelines can help practitioners and patients make decisions in specific contexts. The Grading of Recommendations, Assessment, Development, and Evaluations (GRADE) system has offered a transparent and comprehensive framework for assessing the quality of evidence and the strength of recommendations for systematic reviews and guidelines^11^. The importance of assessing the quality of evidence is to reflect whether the confidence in an estimate of the effect is adequate to support recommendations^12^. Therefore, we conducted a systematic review and meta-analysis by using the GRADE system to evaluate the outcomes of the recurrence rate, complication rate, hospital stay, and drainage duration between VATS operation and conservative treatment during the first episode in patients with PSP.

## Material and methods

We included randomized controlled trials (RCTs) and observational studies following the PICO framework. (1) Population: the first episode of PSP; (2) Intervention: VATS techniques; (3) Comparison: conservative treatment that included observation, intercostal drainage, pigtail drainage, and chest tube drainage; (4) Outcomes: the recurrence rate, complications, hospital stay, and drainage duration. We excluded studies that met at least one of the following criteria: (1) did not directly evaluate treatment outcomes, (2) investigated recurrent, secondary, traumatic, or iatrogenic pneumothorax, (3) did not report both outcomes of VATS and conservative treatment, and (4) involved the duplicate reporting of patient cohorts.

We systematically searched PubMed, EMBASE, Cochrane library, Cochrane Central Register of Controlled Trials, and ClinicalTrials.gov registry electronic databases. The following terms and Boolean operators were used in MeSH and free-text searches: ("pneumothorax"[MeSH Terms] OR "pneumothorax"[All Fields]) AND ("thoracic surgery, video-assisted"[MeSH Terms] OR ("thoracic"[All Fields] AND "surgery"[All Fields] AND "video-assisted"[All Fields]) OR "video-assisted thoracic surgery"[All Fields] OR "vats"[All Fields]). The “related articles” facility in PubMed was used to broaden the search. The search terms and Boolean operators were similar in all databases. Our searches were performed by two experienced reviewers (HYC, YCL) and validated by a certified librarian. No language restrictions were applied. A comprehensive search was performed on August 15, 2020. We searched the reference sections of relevant papers and contacted experts in the field to identify additional studies. We contacted authors if required data were unpublished.

Two researchers independently extracted the data pertaining to participants, inclusion and exclusion criteria, VATS techniques, types of conservative treatment, treatment outcomes, complications, duration of hospital stay, and duration of pleural drainage from the included studies. The independent recorded decisions of the two reviewers were compared, and any disagreements were resolved based on the evaluation of another reviewer.

Two reviewers independently assessed the quality and the risk of bias of the included studies, and the consensus was reached by discussing with other reviewers. We used the Revised Cochrane Risk of Bias tool 2.0 to evaluate the random sequence generation, allocation concealment, blinding of participants and personnel, blinding of the outcome assessment, incomplete outcome data, selective reporting, and other bias in the included RCTs^13^. The Newcastle-Ottawa Quality Assessment Scale was used as the tool to appraise the representativeness, comparability, outcome, and follow-up length of cohort studies^14^.

We assessed each outcome for the quality of evidence by using GRADEpro (GRADEproGDT, http://www.gradepro.org). Factors downgrading the quality included risk of bias, inconsistency, indirectness, imprecision, and publication bias, whereas factors upgrading the quality included large effect, plausible confounding, and dose–response. We classified the quality of evidence as “very low,” “low,” “moderate,” or “high.” We obtained recommendations according to the level of evidence, balancing both desirable and undesirable effects as well as cost-effectiveness and patient preferences^11^. The quality of evidence was assessed by a multidisciplinary team of health care professionals and researchers.

The primary outcomes were the recurrence rate and complications, such as persistent air leaks and surgical complications. Secondary outcomes were the duration of hospital stay and the duration of pleural drainage. We performed subgroup analyses regarding study design, surgical techniques, and types of conservative treatment in the outcome of the recurrence rate and types of complications in the outcome of the complication rate.

We performed statistical analyses by using the Review Manager software version 5.3 (Cochrane Collaboration, Oxford, England, UK) and used Jamovi version 0.9 (free software, https://www.jamovi.org). We performed the meta-analysis based on the Preferred Reporting Items for Systematic Reviews and Meta-analysis (PRISMA) guidelines^15^. The protocol of the study was registered online in the International Prospective Register of Systematic Reviews (PROSPERO: No. CRD42020162267). The effect sizes of dichotomous outcomes were reported as odds ratios (ORs) and Peto odds ratios (ORs), and continuous outcomes were reported as mean differences (MD) with standard deviations (SDs). We used Peto ORs in the meta-analysis with no event in one arm^16^. The precision of effect sizes was reported as a 95% confidence interval (CI). Statistical significance was defined as two-sided *P* values of less than 0.05. We used the random-effects model to calculate the pooled estimate of ORs and mean differences. The random-effect model provides relatively wide CIs and an appropriate estimate of the average treatment effect for statistically heterogeneous studies, contributing a conservative statistical claim^16^. We only pooled data with adequate clinical and methodological similarity. We assessed statistical heterogeneity by using the I^2^ test, which quantified the proportion of the total outcome variability and the variability among studies. To address moderate to high heterogeneity, we also conducted the sensitivity test for meta-analysis results excluding outlying studies^16^. Furthermore, we used the Jamovi software to analyze the funnel plot, Egger’s test, and Begg’s test for the detection of publication bias in the meta-analysis^17^.

## Results

**Figure 1:**
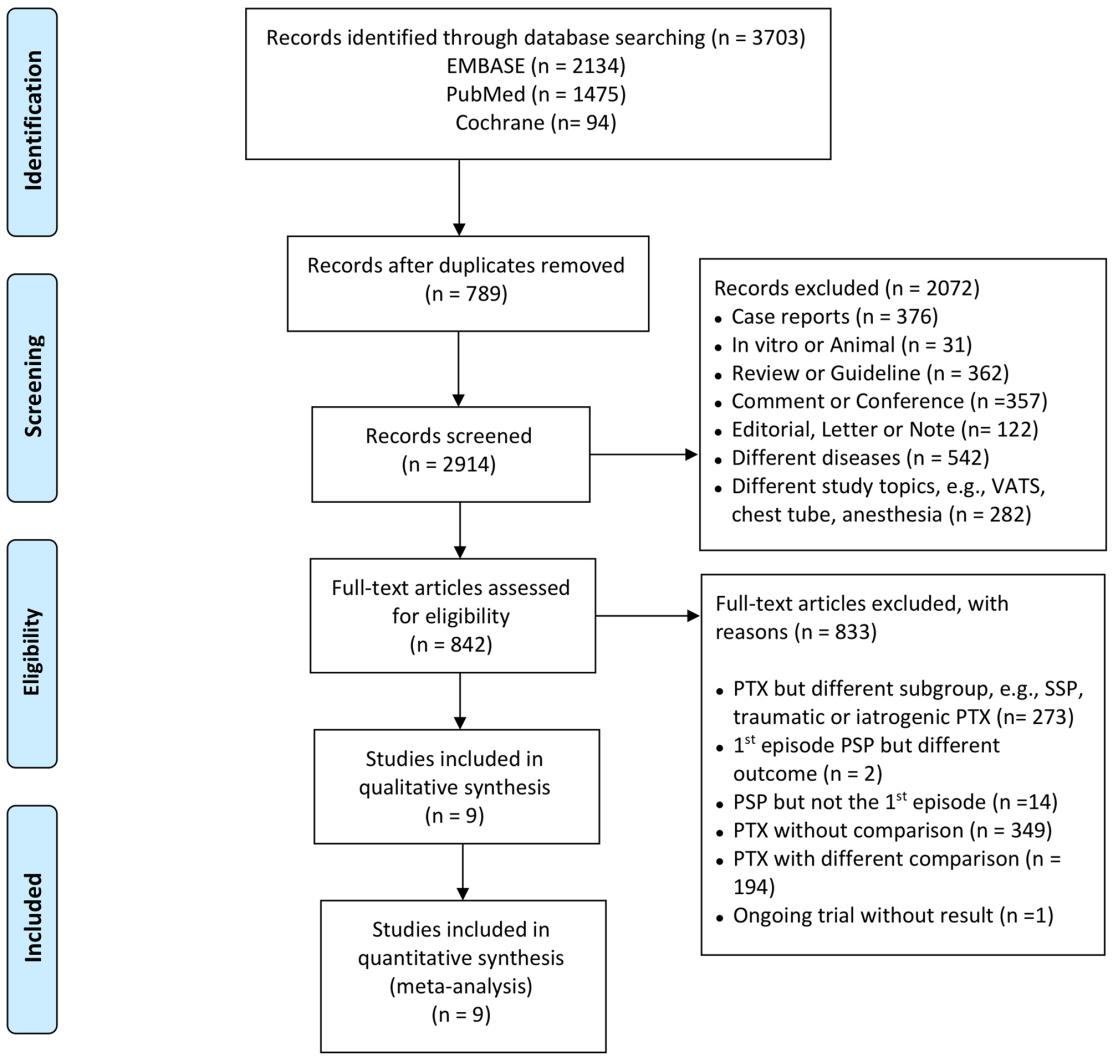
Flow chart of clinical trial selection. The study protocol was conducted in accordance with the Preferred Reporting Items for Systematic Reviews and Meta-Analyses (PRISMA) statement (Moher et al. 2009).

The flowchart in Fig. 1 illustrates the screening and selection process of the study. Our initial search yielded 3703 studies. After excluding duplicates (n = 789), 2914 studies remained. The titles and abstracts of the 2914 studies were screened, and 2072 ineligible studies were excluded. The full text of 842 articles was assessed to determine their eligibility. We excluded 833 citation records for the following reasons: 273 investigated different subgroups of pneumothorax, 349 did not include a comparison group of pneumothorax, 194 investigated a different comparison in patients with pneumothorax, 14 did not investigate the first episode of PSP, two discussed different outcomes in the first episode of pneumothorax, and one trial was terminated on the basis of a poor accrual rate. The remaining 9 eligible studies were included in our meta-analysis^18–26^. The characteristics of eligible studies are presented in Table 1.

**Table 1:** Characteristics of studies that fulfilling the meta-analysis inclusion criteria.

Study	Study design	Nation	Inclusion and exclusion criteria	No. of patients	Age (mean ± SD)	Gender (M/ F)	VATS	Conservative TX
Al-Mourgi [2015]	RCT	Kingdom of Saudi Arabia	Inc: 1st episode of PSP Exc: persistent air leak or unexpanded lung after chest tube insertion, synchronous bil. PTX, hemopneumothorax, tension PTX, recurrence PTX F/U: every 3 months	V/C = 19/22	V/C = 23.8 ± 5.3/22.6 ± 4.8	V: 18/1 C: 20/2	VATS resection of the apical blebs, and apical pleurectomy	Chest tube
Olesen [2018]	RCT	Denmark	Inc: 1st episode of PSP Exc: < 18 y/o; > 40 y/o; previous PTX, SPP, small PTX with only observation, pregnancy, breastfeeding, previous chest surgery, contraindications for anesthesia	V/C = 88/93	V/C = 26.5 ± 6.1/25.8 ± 6.1	V: 69/19 C: 82/11	VATS blebectomy/bullectomy or apical wedge resection of the upper lobe if no blebs/bullae were identified with mechanical pleurodesis in all patients	Chest tube
Divisi [2015]	Pros	Italy	Inc: 1st episode of PSP Exc: acute respiratory insufficiency, no previous lung disease	V/C = 61/61	V/C = 19.6 ± 0.5/22.3 ± 0.8	V: 49/12 C: 46/15	VATS blebectomy/bullectomy or apical wedge resection of the upper lobe if no blebs/bullae were identified with electro-pleurodesis in all patients	Chest tube
Hofmann [2018]	Retros	Germany	Inc: 1st episode PSP; VATS: blebs/bullae in images (n = 20), persistent air leaks > 5 days (n = 15), occupations or personal decision (n = 13)	V/C = 48/87	V/C = 33.1/38.4	V: 34/14 C: 67/20	VATS blebectomy/bullectomy and apical pleurectomy	Chest tube
Iablonskiĭ [2005]	Retros	Russia	Inc: 1st episode of PSP with R. Vanderschuren classification	V/C = 77/115	–	164/28	VATS blebectomy/bullectomy and pleurodesis	Chest tube
Primavesi [2016]	Retros	Austria	Inc: 1st episode of PSP	V/C = 33/23	V/C = 29/23(med)	V: 21/12 C: 18/5	VATS blebectomy/bullectomy in all patients but apical pleurectomy or pleurodesis was added at the surgeon’s discretion	Observation/ oxygen/ chest tube
Sawada [2005]	Retros	Japan	Inc: 1st episode of PSP V group: blebs or bullae positive in CT C group: blebs or bullae negative in CT	V/C = 87/181	9.1 ± 13.6	255/26*	VATS blebectomy/bullectomy in all patients but pleurodesis was performed only in case with multiple bullae and blebs	Observation/ oxygen/ chest tube
Seguier-Lipszyc [2011]	Retros	Israel	Inc: 1st episode of PSP (Age < 18 y/o) Exc: children with underlying lung diseases	V/C = 10/36	16.2	40/6	VATS blebectomy/bullectomy with mechanical and talc pleurodesis in all patients	Oxygen/ chest tube
Soler [2018]	Retros	USA	Inc: 1st episode of PSP Exc: traumatic PTX, not first episode, post-op PTX	V/C = 14/66	V/C = 17.1/17.0	61/20	VATS blebectomy/bullectomy with mechanical pleurodesis in all patients	Observation/ chest tube

*RCT* randomized controlled trial, *pros* prospective, *retros* retrospective, *Inc* inclusion criteria, *Exc* exclusion criteria, *PTX* pneumothorax, *PSP* primary spontaneous pneumothorax, *SPP* secondary spontaneous pneumothorax, *VATS* video-assisted thoracoscopic surgery, *C* conservative treatment, *F/U* follow up, *ER* emergency room, *OR* operating room, *Post-op* post-operative, *med* median.

As listed in Table 1, the 9 studies were published between 2005 and 2018 and included a total of 1121 patients (ranging from 41 to 268 patients). Four studies were from Asia, four from Europe, and one from North America. Of the 9 studies, 2 were RCTs (222 patients)^18,22^, one was a prospective cohort study (122 patients)^19^, and 6 were retrospective cohort studies (777 patients)^20,21,23–26^. Regarding the conservative treatment, 5 studies compared pure chest tube with VATS^18–22^, whereas 4 studies compared observation or chest tube with VATS^23–26^. Surgical techniques used for VATS varied little. Three studies performed VATS with pleurodesis in all surgical patients^22,25,26^, 3 studies conducted VATS with pleurectomy in all surgical patients^18–20^, and 3 studies conducted VATS without regularly performing pleurodesis or pleurectomy^21,23,24^.

Supplementary Table S1 presents the quality assessment of observational studies with the Newcastle-Ottawa Scale for nonrandomized studies. Some studies were determined to have moderate^19,20,23,25,26^ or low^21,24^ methodological quality. Supplementary Table S2 presents the assessment of the risk of bias of 2 RCTs evaluated using ROB 2.0. The methodological quality of the study conducted by Olesen et al.^22^ was of some concern. The study conducted by AI-Mourgi and Alshehri^18^ was evaluated to be of low methodological quality.

**Figure 2:**
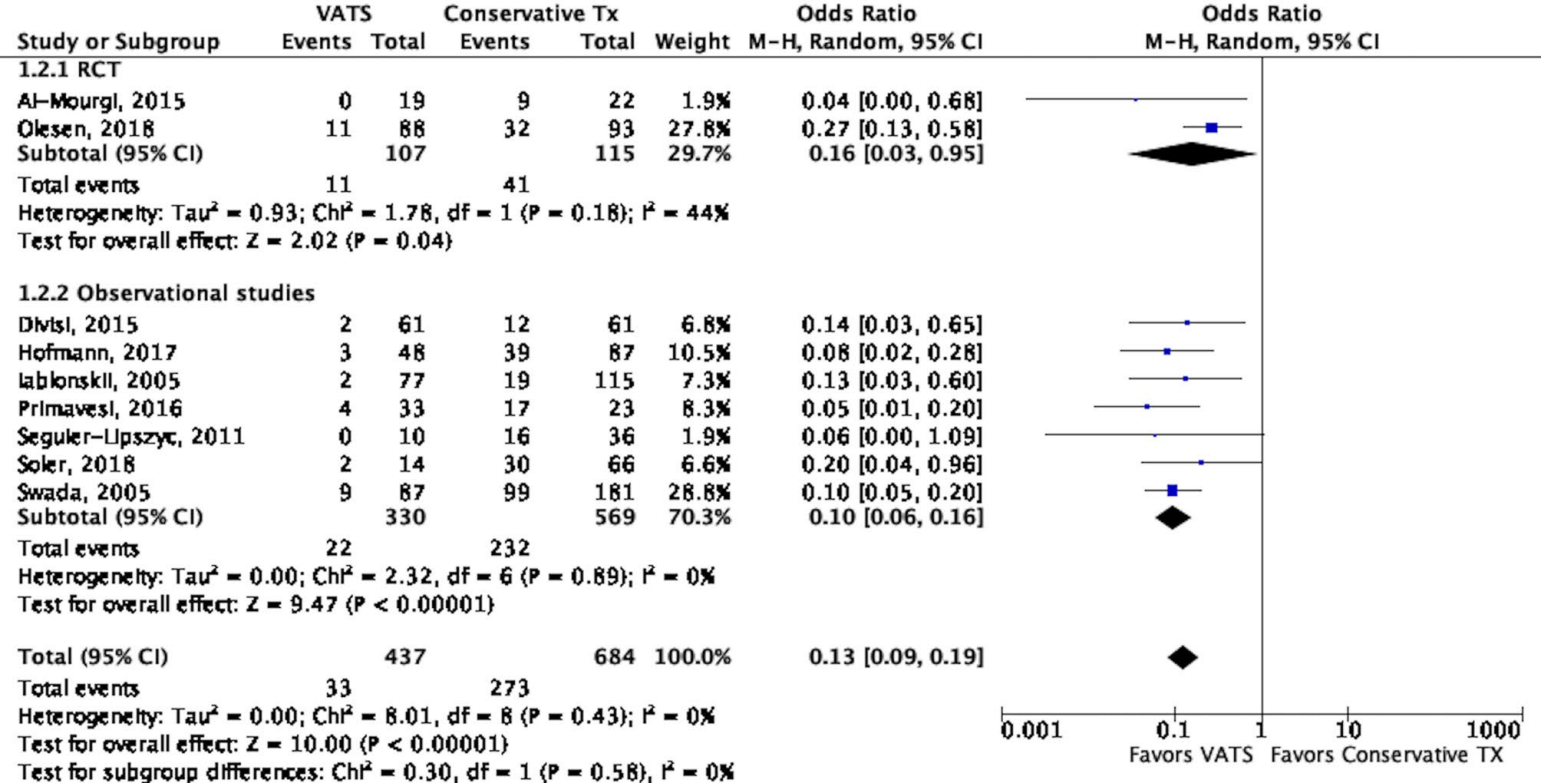
Forest plot of the comparison of recurrence rate between VATS and conservative treatment.

All the studies compared the PSP recurrence rate between conservative treatment and VATS groups. A significant difference was observed in the recurrence rate between groups (OR: 0.13, 95% CI, 0.09 to 0.19, *P* < 0.001) with low heterogeneity (I^2^ = 0%, *P* = 0.43). We determined that the recurrence rate was significantly lower in VATS groups than in conservative treatment groups, both in the subgroup in RCTs (OR: 0.16, 95% CI, 0.03 to 0.95, *P* = 0.04; I^2^ = 44% with *P* = 0.18) and in observation studies (OR: 0.10, 95% CI, 0.06 to 0.16, *P* < 0.001; I^2^ = 0%) (Fig. 2). We performed subgroup analyses regarding types of surgical techniques and types of conservative treatment (Supplementary Fig. S1 and S2). We found that compared with conservative treatment including observation and pleural drainage, recurrence rates were lower for VATS regardless of different types of surgical techniques, such as VATS with pleurodesis in all patients (OR 0.24, 95% CI 0.12–0.46, *P* < 0.001, I^2^ = 0%), VATS with pleurectomy in all patients (OR 0.09, 95% CI 0.04 to 0.23, *P* < 0.001, I^2^ = 0%), or VATS with or without pleurodesis or pleurectomy (OR 0.09, 95% CI 0.05 to 0.16, *P* < 0.001, I^2^ = 0%) (Supplementary Fig. S1). Similarly, recurrence rates were lower for VATS compared with different subgroups of conservative treatment, including the comparisons with conservative treatment with chest tube (OR 0.17, 95% CI 0.09 to 0.29, *P* < 0.001, I^2^ = 3%) or with conservative treatment mixed with observation and chest tube insertion (OR 0.09, 95% CI 0.05 to 0.17, *P* < 0.001, I^2^ = 0%) (Supplementary Fig. S2).

**Figure 3:**
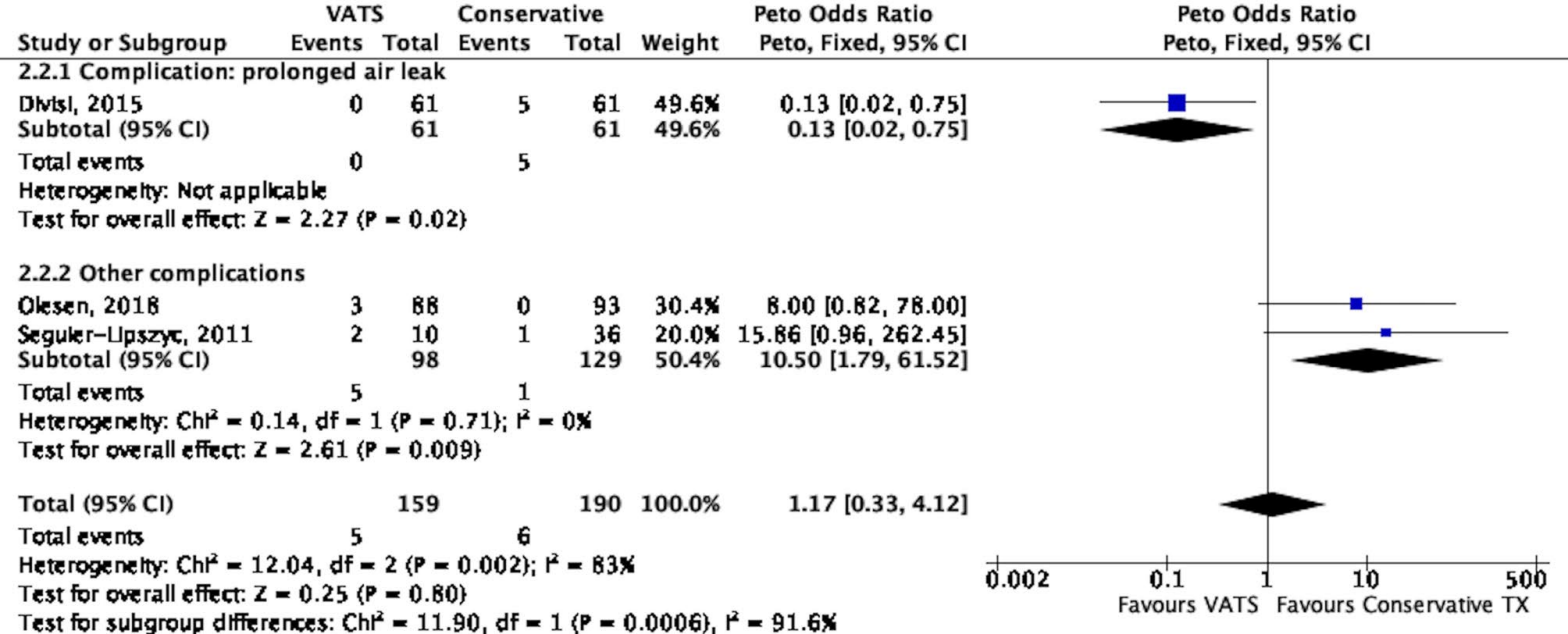
Forest plot of the comparison of complication rate between VATS and conservative treatment.

The incidence of complications between conservative treatment and VATS was reported in 3 studies^19,22,25^. No significant difference in the complication rate was observed (Peto OR 1.17, 95% CI 0.33 to 4.12, *P* = 0.80) between the 2 groups, albeit, heterogeneity was high (I^2^ = 83%, *P* = 0.002) (Fig. 3). Figure 3 showed a subgroup analysis by types of complications. After excluding the study^19^ with the complication of prolonged air leak, we observed that complication rates were significantly higher (Peto OR 10.50, 95% CI 1.79–61.52, *P* = 0.009) in the VATS group than in the conservative treatment group, with low heterogeneity (I^2^ = 0%).

**Figure 4:**
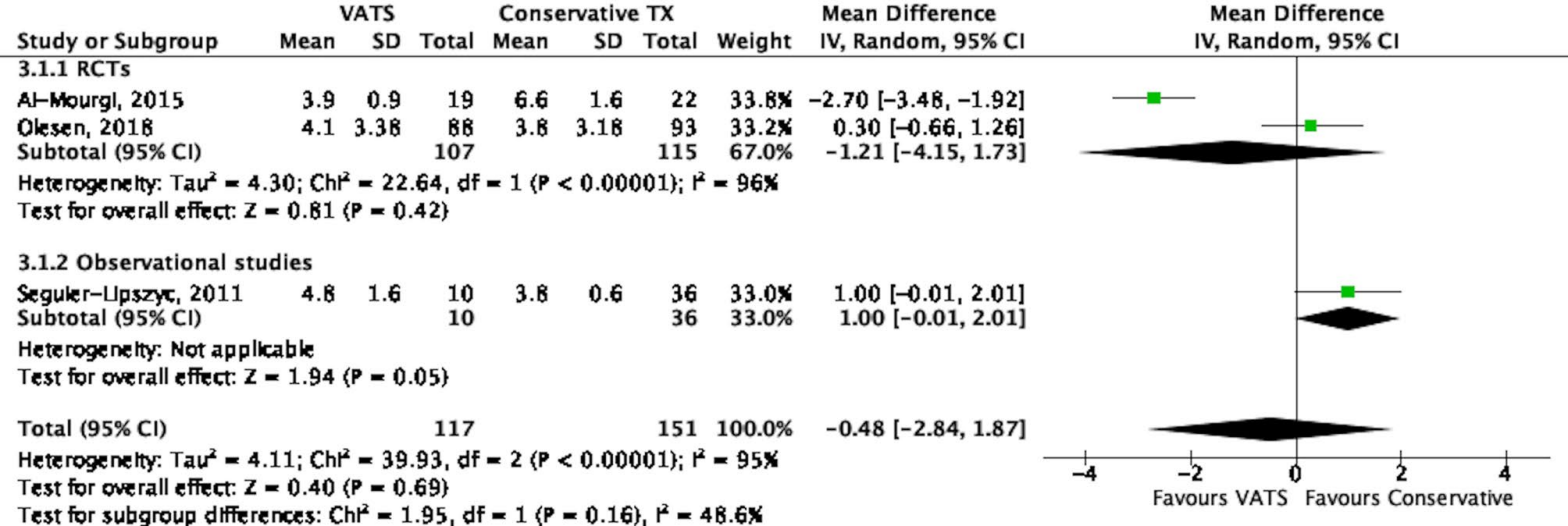
Forest plot of the comparison of duration of hospital stay between VATS and conservative treatment.

Three studies investigated the duration of hospital stay between conservative treatment and VATS groups^18,22,25^. As illustrated in Fig. 4, there was no considerable difference in the duration of hospital stay (MD − 0.48 days, 95% CI − 2.84 to 1.87, *P* = 0.69) between the 2 groups. However, significant heterogeneity (I^2^ = 95%, *P* < 0.001) across studies was observed. To identify the source of heterogeneity, we performed the sensitivity test (Supplementary Fig. S3. After excluding Al-Mourgi’s study^18^, our result suggested that hospital stay did not differ between conservative treatment and VATS groups (MD 0.63 days, 95% CI − 0.06 to 1.33, *P* = 0.08; I^2^ = 0%).

**Figure 5:**
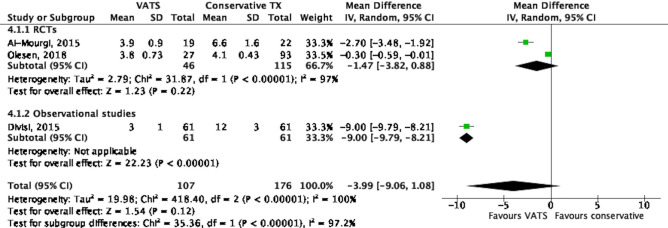
Forest plot of the comparison of drainage duration between VATS and conservative treatment.

As displayed in Fig. 5, the duration of pleural drainage was reported in 3 studies^18,19,22^. There was no difference in the duration of pleural drainage between conservative treatment with pleural drainage and VATS groups, with a mean difference of − 3.99 days (95% CI − 9.06 to 1.08, *P* = 0.12). However, heterogeneity across studies was high (I^2^ = 100%, *P* < 0.001).

Finally, based on the asymmetry of the funnel plot for the recurrence rate, we did not find significant unbalance in this meta-analysis (Supplementary Fig. S4). Results were similar by using Egger’s (*P* = 0.218) and Begg’s (*P* = 0.919) tests, which suggested that there was no significant publication bias in our meta-analysis.

Table 2 presents the GRADE approach for rating the quality of evidence. The recurrence rate and bleeding complication were judged as critical outcomes, and complications involving prolonged air leak, hospital stay, and drainage duration were considered as important outcomes, which were discussed by our team group. The process of rating the quality of evidence began with the study design (RCT trial or observational studies)^27,28^. Thus, we stratified the analysis according to RCTs and observation studies for each outcome (recurrence rate, hospital stay, and drainage duration). The quality of evidence of the recurrence rate in both RCTs and observational studies was rated as moderate. The RCTs were rated down from high to moderate for the recurrence rate outcome because of the high risk of bias, whereas the observational studies were rated up from low to moderate owing to very large effects (OR 0.10, 95% CI 0.06 to 0.16, *P* < 0.001; I^2^ = 0%) across studies. The evidence of the bleeding complication was rated as moderate owing to imprecision, which indicated that the clinical decision would differ because of the upper or lower boundaries of the CI (OR 8, 95% CI 0.82 to 78). The evidence of complications of prolonged air leak was rated from low to moderate owing to a very large effect size (OR 0.13, 95% CI 0.02 to 0.75, *P* = 0.02). The quality of evidence in the individual outcomes of hospital stay and drainage duration were both judged as very low because of the high risk of bias, inconsistency, and imprecision. An overall rating of confidence in the estimates of effect is based on the critical outcome that provides the lowest confidence^29^. Hence, we made an overall rating of confidence in effect estimates as moderate based on critical outcomes (recurrence rate and bleeding complication).

**Table 2:** VATS compared to conservative treatment for the first episode of primary spontaneous pneumothorax.

Certainty assessment							No. of patients		Effect		Certainty	Importance
No. of studies	Study design	Risk of bias	Inconsistency	Indirectness	Imprecision	Other considerations	VATS	Conservative treatment	Relative (95% CI)	Absolute (95% CI)		
**Recurrence rate**												
2	Randomized trials	Serious^a^	Not serious	Not serious	Not serious	None	11/107 (10.3%)	41/115 (35.7%)	OR 0.16 (0.03 to 0.95)	275 fewer per 1000 (from 340 to 12 fewer)	⨁⨁⨁◯ Moderate	Critical
7	Observational studies	Serious^b^	Not serious	Not serious	Not serious	Very strong association^f^	22/330 (6.7%)	232/569 (40.8%)	OR 0.10 (0.06 to 0.16)	343 fewer per 1000 (from 368 to 309 fewer)	⨁⨁⨁◯ Moderate	Critical
**Complication: bleeding**												
1	Randomized trials	Not serious	Not serious	Not serious	Serious^e^	None	3/88 (3.4%)	0/93 (0.0%)	OR 8.00 (0.82–78.00)	36 more per 1000 (from 1 fewer to 291 more)	⨁⨁⨁◯ Moderate	Critical
**Complication: prolonged air leak**												
1	observational studies	Serious^b^	Not serious	Not serious	Not serious	Very strong association^g^	0/61 (0.0%)	5/61 (8.2%)	OR 0.13 (0.02–0.75)	70 fewer per 1000 (from 80 to 19 fewer)	⨁⨁⨁◯ Moderate	Important
**Hospital stay**												
2	Randomized trials	Serious^a^	Serious^c^	Not serious	Not serious	None	107	115	–	MD 1.21 days lower (4.15 lower to 1.73 higher)	⨁⨁◯◯ Low	Important
1	Observational studies	Serious^b^	Not serious	Not serious	Not serious	None	10	36	–	MD 1 days higher (0.01 lower to 2.01 higher)	⨁◯◯◯ Very low	Important
**Drainage duration**												
2	Randomized trials	Serious^a^	Serious^d^	Not serious	Not serious	None	46	115	–	MD 1.47 days lower (3.82 lower to 0.88 higher)	⨁⨁◯◯ Low	Important
1	Observational studies	Serious^b^	Not serious	Not serious	Not serious	None	61	61	–	MD 9 days lower (9.79 lower to 8.21 lower)	⨁◯◯◯ Very low	Important

Factors downgrading the quality include risk of bias, inconsistency, indirectness, imprecision, and publication bias, whereas factors upgrading the quality include large effect, plausible confounding, and dose–response. The quality of evidence is classified as “very low,” “low,” “moderate,” or “high” .

*CI* confidence interval, *OR* Odds ratio, *MD* mean difference.

^a^Risk of bias: Al-Mourgi et al. (2015): high ROB.

^b^Risk of bias: Divisi et al. (2015) and Seguier-Lipszyc et al. (2011): high ROB.

^c^Inconsistency: hospital stay I^2^: 96%.

^d^Inconsistency: duration drainage I^2^: 97%.

^e^Imprecision: the clinical decision would differ due to the upper or the lower boundaries of the CI [OR 8, 95% CI 0.82 to 78], which represented the surgical bleeding.

^f^The effect was very large due to the OR is 0.10 which is less than 0.2.

^g^The effect was very large due to the OR is 0.13 which is less than 0.2.

## Discussion

Although international guidelines do not advocate surgery for patients with the first episode of pneumothorax, except in cases of complicated pneumothorax or for occupations at risk^3,4,10^. The optimal management of these patients has remained debatable^30,31^. Therefore, we conducted a systematic review and meta-analysis to facilitate the integration of information from current trials and to assist clinicians in making treatment decisions. We employed the GRADE system for a more transparent and efficient rating of the quality of evidence and the strength of recommendations. We included two RCTs and seven observational studies in the meta-analysis^18–26^. Our results demonstrated that patients with the first episode of PSP would have a significant reduction of the ipsilateral recurrence rate when treated with VATS than when treated with conservative treatment. However, the VATS group also had a higher rate of bleeding complications than conservative treatment, despite not being statistically significant. In addition, we found no difference regarding hospital stay and drainage duration between VATS and conservative treatment groups, but the quality of current evidence for these two outcomes was very low.

We used the GRADE system to evaluate the quality of evidence because it evaluates more than just the risk of bias, which refers to an appraisal of the internal validity of an individual study^28^. The quality of evidence according to the GRADE system also reflects the degree of confidence of an estimate of effect for systematic reviews^12^. Therefore, we regard the strength of recommendation of performing VATS surgery for the first episode of PSP as weak because the evidence is of moderate (recurrence rate and bleeding complication) to very low quality (hospital stay and drainage duration) in individual outcomes, and the desirable effect (recurrence rate) does not outweigh undesirable effects (bleeding complication, total costs, hospital stay, and drainage duration). Furthermore, we suggest sharing the decision-making for patients with the first episode of PSP in order to reach the optimal personal decision, which incorporates patient preferences and values, balances both desirable and undesirable effects, and cost-effectiveness. It is crucial for clinicians to recognize that different treatment options will be suitable for different patients and thus help patients reach a decision according to their preferences and values. Therefore, we offer a weak recommendation for these circumstances.

Daemen et al. conducted a systematic review and meta-analysis comparing chest tube drainage versus VATS for the first episode of PSP by analyzing two randomized trials and two observational studies^32^. They found that VATS could significantly reduce the recurrence rate and duration of hospital stay. In our study, we incorporated whole conservative treatments, which typically comprised observation, aspiration, thoracentesis, and chest tube drainage, to have a broader application in clinical settings. We also performed subgroup analysis by stratifying the analysis according to methods of conservative treatment (Supplementary Fig. S2). We searched thoroughly without language limitation and included nine studies, but we did not find any studies comparing sole observation or sole aspiration with VATS. Our results showed that the recurrence rate in the VATS group was significantly lower than in the subgroup with chest tube only and the subgroup with observation and chest tube. Furthermore, we employed the GRADE system to investigate the quality of evidence to inform recommendations, which is practicable and essential for clinical practice.

Our findings showed that the recurrence rate was significantly lower after VATS than after conservative treatment. The quality of evidence was moderate; thus, further research will probably have a considerable effect on our confidence in the estimate of effect and may also change the estimate^12^. The number needed to treat (NNT) in our study was 3.1, which indicates that for every three patients that undergo VATS operations, one recurrence is avoided. Although the results suggested VATS was associated with lower recurrence rates, not all patients with the first episode of PSP should undergo VATS operations, because there is a concern of overtreatment for two-thirds of patients. Hoffman et al. reported that nearly 50% of patients healed without recurrence with conservative treatments, and the overtreatment rate of pneumothorax patients was nearly 60%^20^. Although VATS is associated with a very low operative risk, surgical trauma, and complication rate, surgeons still need to explore safe and effective adhesion therapy, which is a mild and spotty adhesion that can prevent the collapse of the lungs. Recurrence rates can be significantly reduced by strong and tight adhesion. However, it is important for clinicians to consider that excessively tight adhesion might increase risks associated with future chest surgeries in people who have undergone pleurodesis or pleurectomy^31^.

Although no significant difference was observed in the pooled data of complication rates between conservative and VATS groups, the heterogeneity was high. Therefore, we separated subgroups to prolonged air leaks and other complications, which effectively reduced the heterogeneity. Prolonged air leak has been reported as one of the most common complications of pleural drainage in pneumothorax patients^19,33,34^. We also found that VATS groups have higher rates of complications than conservative groups. Olsen et al. reported that three surgical patients underwent reintervention because of bleeding from intercostal arteries^22^. Although the complication of bleeding was not significantly different between groups, our clinical decision would differ because the upper CI represented the surgical bleeding. The other complications reported included re-expansion edema in the pleural drainage group and postoperative complications, one with fever and one with phlebitis, in the VATS group^25^. The risk of complications from VATS should not be overlooked despite it being less invasive than open surgery. It is important that patients understand the possibility of complications from different treatments before making a decision.

We found no significant differences in the duration of hospital stay and the drainage duration between conservative and VATS groups. Only three studies were included in outcomes of hospital stay and drainage duration. Furthermore, we rated those outcomes as having high heterogeneity and very low quality of evidence by using the GRADE system, which indicates that the estimate of effect is very uncertain. The drainage duration can be influenced both by external factors (e.g., physicians’ decisions, and hospitals’ routine practices) and internal factors (e.g., lung conditions and initial severity of pneumothorax at diagnosis). Thus far, neither CHEST^10^ nor the British Thoracic Society^4^ has provided explicit recommendations concerning the timing of removal of intercostal drains, which may result in different standards on when to remove these drains and the timing of discharge. To minimize the effect of external factors (e.g., physicians’ decisions and hospitals’ practices), we need clear guidelines and more studies with large patient numbers to investigate these issues.

The economic outcome plays a critical role in robust clinical guidelines and recommendations. However, only one included study^19^ reported that the cost of VATS was more advantageous than that of pleural drainage (2423 vs 4855 Euros). Other studies have reported that the costs of VATS were higher than those of conservative treatment for patients with pneumothorax^6,33,35^ and the contrary^36^. Those studies did not meet our inclusion criteria, such as populations of combined PSP and SSP or recurrent pneumothorax. Therefore, further research of economic evaluations such as costs, resource use, and affordability are warranted.

There are some limitations to our study. First, because we did not have individual patient data (IPD), we could not perform stratified analyses according to age, sex, BMI, smoking status, initial severity of pneumothorax, or radiographic findings that might indicate the risk of recurrence. Second, we only included two RCTs and seven observational studies in our meta-analysis. The outcomes of complications, hospital stay, and drainage duration were not assessed in all nine studies, and the quality of evidence in those outcomes was very low. Furthermore, only one included study^19^ reported on economic evaluation, which was insufficient for meta-analysis. To assess potential bias due to the various qualities of the included studies and heterogeneity, we incorporated the quality assessment of included studies (Supplementary Table S1 and S2) and the quality of evidence by using the GRADE system (Table 2). We also conducted subgroup and sensitivity analyses (Supplementary e-Figure S1, S2 and S3). Third, we did not include the outcomes of quality of life or pain score because studies regarding those evaluations were few and unstandardized^19,37,38^. Fourth, as the case for most meta-analyses, publication bias is a potential concern, which can affect the validity and generalizability of the findings. Nonetheless, we conducted a comprehensive search of available literature to minimize the possibility of publication bias. We also conducted the funnel plot (Supplementary e-Figure S4) and the corresponding statistics of Egger’s and Begg’s tests, which suggested no significant publication bias. However, given the limited studies, we encouraged an updated analysis once several other studies become available. Further research is needed for the grading of recommendation, as it includes the continuum of quality of evidence. Outcomes such as complications, hospital stay, drainage duration, economic evaluation, quality of life, and pain score between conservative and VATS groups will necessitate clarification to facilitate decision-making in clinical settings.

We offer a weak recommendation regarding VATS as the treatment for the first episode of PSP according to our systematic review of the current evidence using the GRADE system. Weak recommendation indicates that clinicians should recognize that different choices will be appropriate for different patients and that they must help each patient reach a treatment decision consistent with their values and preferences through shared decision making.

## Supplementary Information

Supplementary Information 1.

Supplementary Information 2.

## Abbreviations

PSP: Primary spontaneous pneumothorax
SSP: Secondary spontaneous pneumothorax
PTX: Pneumothorax
VATS: Video-assisted thoracoscopic surgery
GRADE system: Grading of Recommendations, Assessment, Development, and Evaluations system
RCTs: Randomized controlled trials
ROB: Risk of Bias
PRISMA: Preferred Reporting Items for Systematic Reviews and Meta-analysis
MD: Mean difference
ORs: Odds ratios
SDs: Standard deviations
CI: Confidence interval
NNT: Number needed to treat
BMI: Body mass index
MeSH: Medical Subject Headings
