# Supplementary material for: Recommendation for management of patients with their first episode of primary spontaneous pneumothorax, using video-assisted thoracoscopic surgery or conservative treatment
Source: Sci Rep. 2021 May 25;11:10874. doi: 10.1038/s41598-021-90113-w (PMC8149688; doi:10.1038/s41598-021-90113-w)
Supplement: Supplementary file 1 — Supplementary Information 1. [file 41598_2021_90113_MOESM1_ESM.pdf]

Title: Recommendation for management of patients with their first episode of primary spontaneous pneumothorax, using video-assisted thoracoscopic surgery or conservative treatment

Hsin-Yi Chiu, MD, Yi-Chia Ho, MD, Pei-Chen Yang, MD, Chi-Ming Chiang, MD, Cheng-Chin Chung, MD,Wei-Ciao Wu, MD, Yu-Cih Lin, RN, PhD, Chien-Yu Chen, MD, PhD, Yu-Chung Wu, MD

Table S1 Quality assessment with the Newcastle-Ottawa Quality Assessment Scale

Study	Selection				Comparability	Outcome			Quality Scores
	Item 1	Item 2	Item 3	Item 4	Item 5	Item 6	Item 7	Item 8	
**Divisi [2015]**	★	★	★	★		★	★	★	7
**Hofmann [2018]**	★	★	★		★	★	★	★	7
**Iablonskiĭ [2005]**	★	★	★			★	★	★	6
**Primavesi [2016]**	★	★	★		★	★	★	★	7
**Sawada [2005]**	★	★	★			★		★	5
**Seguier-Lipszyc [2011]**	★	★	★		★	★	★	★	7
**Soler [2018]**	★	★	★		★	★	★	★	7

Item 1: Representativeness of the exposed cohort
Item 2: Selection of the non-exposed cohort
Item 3: Ascertainment of exposure
Item 4: Demonstration that outcome of interest was not present at start of study
Item 5: Comparability of cohorts on the basis of the design or analysis controlled for confounders
Item 6: Assessment of outcome
Item 7: Was follow-up long enough for outcomes to occur
Item 8: Adequacy of follow-up of cohorts
Total scores: 9 (maximum)

Table S2. Randomized controlled trials evaluated using the revised Cochrane Risk of Bias (ROB 2.0) tool

**Author, year**	**Al-Mourgi [2015]**	**Olesena [2018]**
**Bias arising from the randomization process**	Some concerns	Low
**Bias due to deviations from the intended interventions**	Some concerns	Some concerns
**Bias due to missing outcome data**	Some concerns	Low
**Bias in measurement of the outcome**	Low	Low
**Bias in selection of the reported result**	Some concerns	Low
Overall bias	*High*	*Some concerns*

e-Figure S1

Study or Subgroup	VATS Events	VATS Total	Conservative Tx Events	Conservative Tx Total	Weight	Odds Ratio M-H, Random, 95% CI
**1.3.1 VATS with pleurodesis in all patients**						
Olesen, 2018	11	88	32	93	27.8%	0.27 [0.13, 0.58]
Seguier-Lipszyc, 2011	0	10	16	36	1.9%	0.06 [0.00, 1.09]
Soler, 2018	2	14	30	66	6.6%	0.20 [0.04, 0.96]
Subtotal (95% CI)		112		195	36.3%	0.24 [0.12, 0.46]
Total events	13		78			
Heterogeneity: $Tau^2 = 0.00$; $Chi^2 = 1.09$, df = 2 (P = 0.58); $I^2 = 0\%$						
Test for overall effect: Z = 4.21 (P < 0.0001)						
**1.3.2 VATS with pleurectomy in all patients**						
Al-Mourgi, 2015	0	19	9	22	1.9%	0.04 [0.00, 0.68]
Divisi, 2015	2	61	12	61	6.8%	0.14 [0.03, 0.65]
Hofmann, 2017	3	48	39	87	10.5%	0.08 [0.02, 0.28]
Subtotal (95% CI)		128		170	19.2%	0.09 [0.04, 0.23]
Total events	5		60			
Heterogeneity: $Tau^2 = 0.00$; $Chi^2 = 0.69$, df = 2 (P = 0.71); $I^2 = 0\%$						
Test for overall effect: Z = 5.11 (P < 0.00001)						
**1.3.3 VATS with or without pleurodesis/pleurectomy**						
Iablonskii, 2005	2	77	19	115	7.3%	0.13 [0.03, 0.60]
Primavesi, 2016	4	33	17	23	8.3%	0.05 [0.01, 0.20]
Swada, 2005	9	87	99	181	28.8%	0.10 [0.05, 0.20]
Subtotal (95% CI)		197		319	44.5%	0.09 [0.05, 0.16]
Total events	15		135			
Heterogeneity: $Tau^2 = 0.00$; $Chi^2 = 1.06$, df = 2 (P = 0.59); $I^2 = 0\%$						
Test for overall effect: Z = 7.84 (P < 0.00001)						
Total (95% CI)		437		684	100.0%	0.13 [0.09, 0.19]
Total events	33		273			
Heterogeneity: $Tau^2 = 0.00$; $Chi^2 = 8.01$, df = 8 (P = 0.43); $I^2 = 0\%$						
Test for overall effect: Z = 10.00 (P < 0.00001)						
Test for subgroup differences: $Chi^2 = 5.19$, df = 2 (P = 0.07), $I^2 = 61.5\%$						

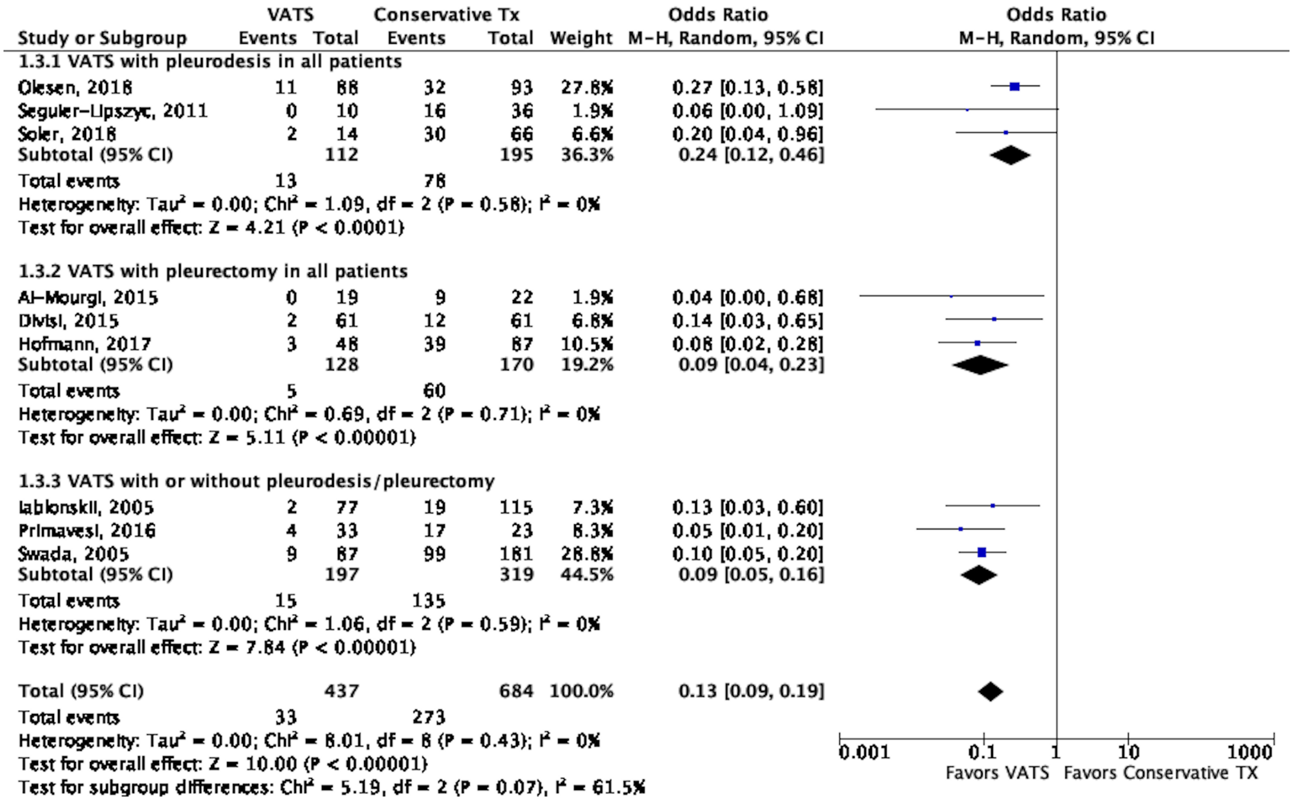

e-Figure S2

Study or Subgroup	VATS Events	VATS Total	Conservative Tx Events	Conservative Tx Total	Weight	Odds Ratio M-H, Random, 95% CI
**1.4.1 Conservative treatment with chest tube**						
Al-Mourgi, 2015	0	19	9	22	1.9%	0.04 [0.00, 0.68]
Divisi, 2015	2	61	12	61	6.8%	0.14 [0.03, 0.65]
Hofmann, 2017	3	48	39	87	10.5%	0.08 [0.02, 0.28]
Iablonskii, 2005	2	77	19	115	7.3%	0.13 [0.03, 0.60]
Olesen, 2018	11	88	32	93	27.8%	0.27 [0.13, 0.58]
Subtotal (95% CI)		293		378	54.4%	0.17 [0.09, 0.29]
Total events	18		111			
Heterogeneity: Tau² = 0.02; Chi² = 4.13, df = 4 (P = 0.39); I² = 3%						
Test for overall effect: Z = 6.25 (P < 0.00001)						
**1.4.2 Conservative treatment with observation or chest tube**						
Primavesi, 2016	4	33	17	23	8.3%	0.05 [0.01, 0.20]
Seguier-Lipszyc, 2011	0	10	16	36	1.9%	0.06 [0.00, 1.09]
Soler, 2018	2	14	30	66	6.6%	0.20 [0.04, 0.96]
Swada, 2005	9	87	99	181	28.8%	0.10 [0.05, 0.20]
Subtotal (95% CI)		144		306	45.6%	0.09 [0.05, 0.17]
Total events	15		162			
Heterogeneity: Tau² = 0.00; Chi² = 1.83, df = 3 (P = 0.61); I² = 0%						
Test for overall effect: Z = 7.84 (P < 0.00001)						
**Total (95% CI)**		437		684	100.0%	0.13 [0.09, 0.19]
Total events	33		273			
Heterogeneity: Tau² = 0.00; Chi² = 8.01, df = 8 (P = 0.43); I² = 0%						
Test for overall effect: Z = 10.00 (P < 0.00001)						
Test for subgroup differences: Chi² = 1.95, df = 1 (P = 0.16), I² = 48.8%						

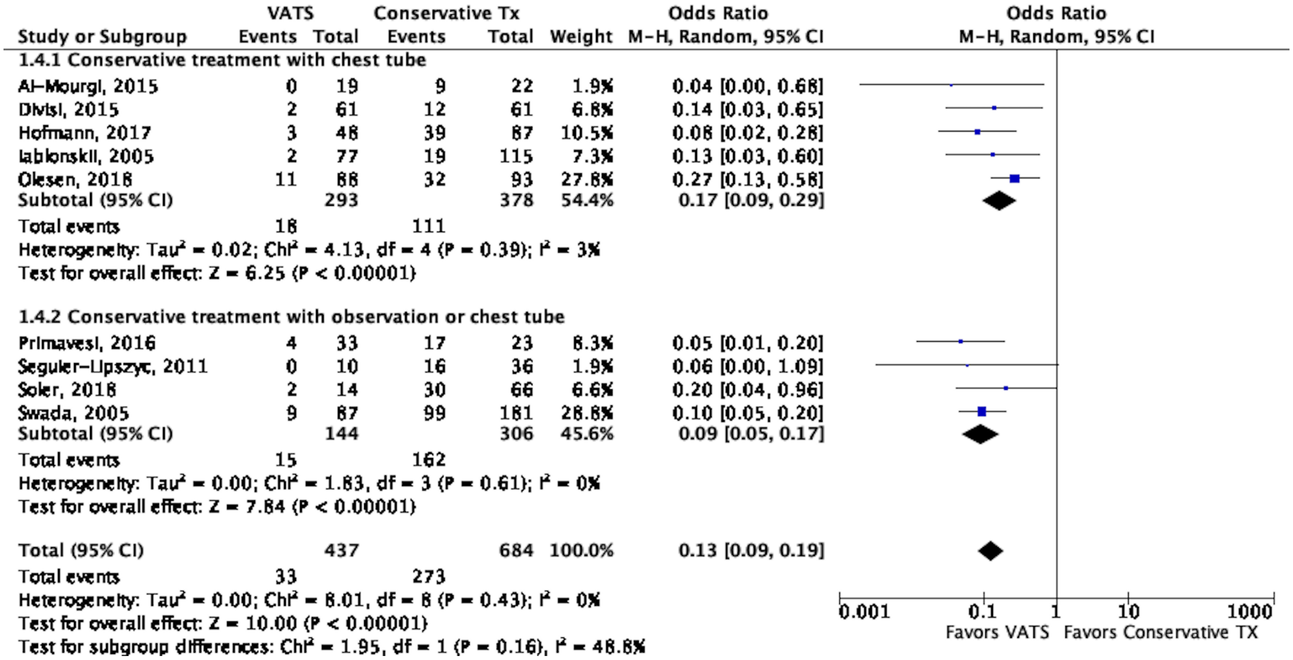

e-Figure S3

Study or Subgroup	VATS Mean	VATS SD	VATS Total	Conservative TX Mean	Conservative TX SD	Conservative TX Total	Weight	Mean Difference IV, Random, 95% CI
Al-Mourgi, 2015	3.9	0.9	19	6.6	1.6	22	0.0%	−2.70 [−3.48, −1.92]
Olesen, 2018	4.1	3.38	88	3.8	3.18	93	52.7%	0.30 [−0.66, 1.26]
Seguier-Lipszyc, 2011	4.8	1.6	10	3.8	0.6	36	47.3%	1.00 [−0.01, 2.01]
Total (95% CI)			98			129	100.0%	0.63 [−0.06, 1.33]

Heterogeneity: $Tau^2 = 0.00$; $Chi^2 = 0.97$, df = 1 (P = 0.32); $I^2 = 0\%$
Test for overall effect: Z = 1.78 (P = 0.08)

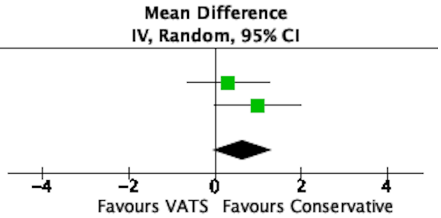

e-Figure S4

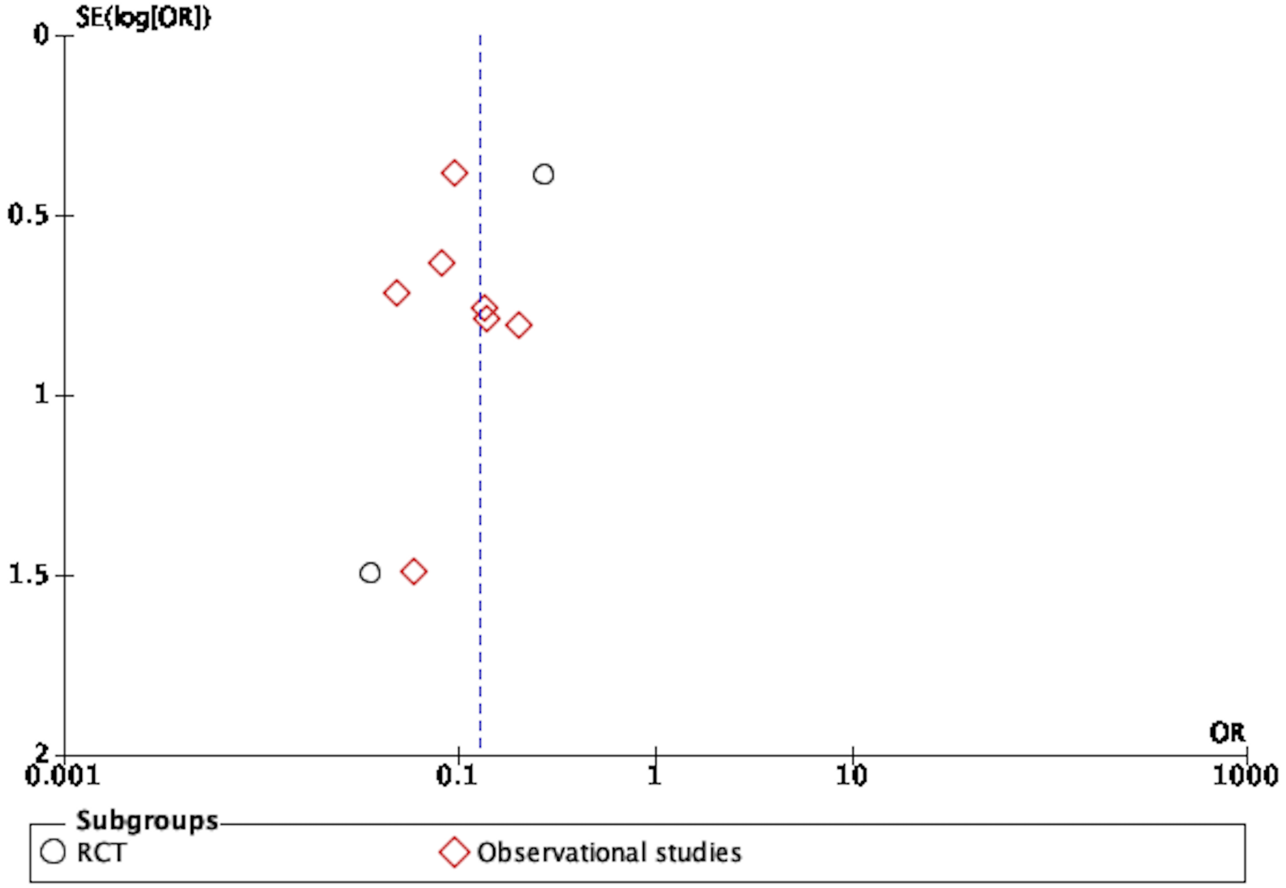

SE(log[OR])
0
0.5
1
1.5
2
OR
0.001
0.1
1
10
1000
Subgroups
RCT
Observational studies
