# Supplementary material for: Recommendation for management of patients with their first episode of primary spontaneous pneumothorax, using video-assisted thoracoscopic surgery or conservative treatment
Source: Sci Rep. 2021 May 25;11:10874. doi: 10.1038/s41598-021-90113-w (PMC8149688; doi:10.1038/s41598-021-90113-w)
Supplement: Supplementary file 2 — Supplementary Information 2. [file 41598_2021_90113_MOESM2_ESM.docx]

e-Figure S1. Forest plot of the comparison of recurrence rate between VATS and conservative treatment with subgroup analysis by types of surgical techniques

e-Figure S2. Forest plot of the comparison of recurrence rate between VATS and conservative treatment with subgroup analysis by types of conservative treatment

e-Figure S3. Forest plot of the comparison of duration of hospital stay between VATS and conservative treatment with sensitivity test by excluding AI-Mourgi’s study

e-Figure S4. Funnel plot of the meta-analysis of published studies
